# The Role of Inflammation in CKD

**DOI:** 10.3390/cells12121581

**Published:** 2023-06-07

**Authors:** Saurav Prashant Kadatane, Matthew Satariano, Michael Massey, Kai Mongan, Rupesh Raina

**Affiliations:** 1 Department of Medicine, Northeast Ohio Medical University, Rootstown, OH 44272, USA; skadatane@neomed.edu (S.P.K.);; 2 Akron Nephrology Associates/Cleveland Clinic Akron General Medical Center, Akron, OH 44302, USA; 3 Department of Nephrology, Akron Children’s Hospital, Akron, OH 44308, USA

**Keywords:** chronic kidney disease, inflammation, immune

## Abstract

Chronic kidney disease (CKD) affects many adults worldwide. Persistent low-grade inflammation is a substantial factor in its development and progression and has correlated with increased mortality and cardiovascular problems. This low-grade inflammation is a product of dysregulation of the normal balance between pro- and anti-inflammatory markers. Various factors such as increased innate immune system activation, reactive oxygen species production, periodontal disease, dysregulation of anti-inflammatory systems and intestinal dysbiosis result in the dysregulation of this balance. Furthermore, this low-grade inflammation has down-effects such as hypertension, renal fibrosis and acceleration of renal function decline. Moreover, low-grade inflammation over time has been linked to malignancy in CKD. As CKD progresses, many patients require dialysis, which has a negative bidirectional relationship with persistent inflammation. Treatment options for inflammation in CKD are vast, including cytokine inhibitors, statins and diets. However, more research is needed to create a standardized management plan. In this review, we will examine the normal physiology of the kidney and its relationship with the immune system. We will then delve into the pathology behind persistent inflammation, the various causes of inflammation, the downstream effects of inflammation, dialysis and potential treatments for inflammation in CKD.

## 1. Introduction

**Figure 1:**
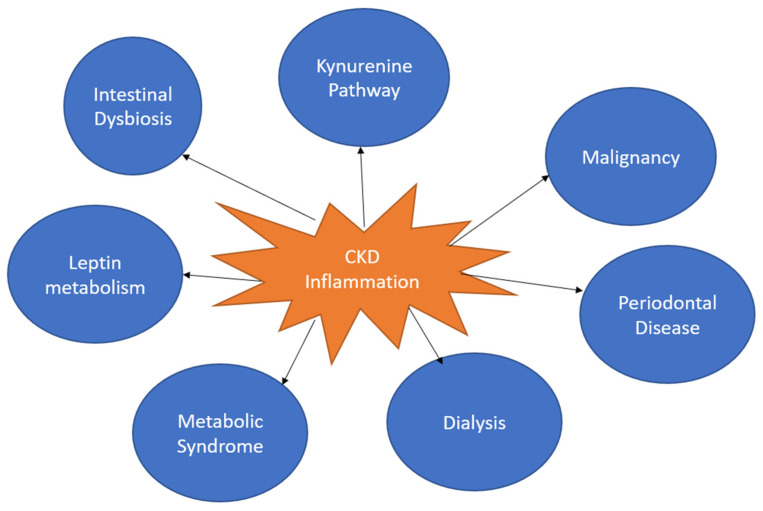
This figure shows the various aspects of inflammation in CKD.

Chronic kidney disease (CKD), an abnormal structure or function present for over 3 months, affects around 14% of US adults [1,2]. There is often an increased inflammatory state in patients with CKD. Low-grade systemic inflammation also is a substantial contributor to the development of CKD. However, the exact timeline between the initiation of inflammation and CKD is unclear. Low-grade systemic inflammation is a constant presence of inflammatory markers. Many conditions are associated with low-grade inflammation, such as metabolic syndrome, non-alcoholic fatty liver disease, cardiovascular disease and diabetes [3]. The inflammatory cascade is characterized by an inciting stimulus of tissue injury or foreign entity increasing the generation of proinflammatory cytokines (e.g., TNF-alpha and IL-1), resulting in increased blood flow, upregulation of chemical mediators and leukocyte infiltration [4]. The discontinuation of this cascade is mediated by anti-inflammatory molecules. However, low-grade inflammation can persist when there is a failure of discontinuation or persistence of proinflammatory markers. The persistence of an inflammatory state may be caused by poor diet and nutrition. Poor gut microbiota, maternal health during pregnancy, childhood infections and stress have all been linked with an inflammatory response [5]. CKD creates a setting for many causes of an increased inflammatory state, such as uremia, oxidative stress, infections, dyslipidemia, malnutrition, volume overload, dialysis, periodontal disease and reduced clearance of inflammatory factors (Figure 1) [6]. The unique physiology of the kidneys also puts them at a greater risk for inflammation-related damage [2]. This review will examine the various causes of persistent inflammatory states, the pathophysiology of chronic inflammatory states, the unique effects of inflammation on the kidneys and the relationship to chronic kidney disease.

## 2. Renal Physiology and Inflammation

### 2.1. Protective Measures against Inflammation in Kidneys

The kidneys play a large role in maintaining homeostasis of the immune system. The kidneys are responsible for clearing cytokines and bacterial antigens from circulation. Removal of proinflammatory cytokines and pathogen-associated molecular patterns (PAMPs) can reduce inflammation and activation of immune cells [7]. In addition to clearance, the kidneys play an important role in peripheral tolerance through resident dendritic cells (DCs) and macrophages (Mac) of the M2 subtype. DCs and Mac have been shown to inhabit the kidneys’ glomerular compartment, cortical and peritubular interstitium [8]. As the nephron filtrate is reabsorbed, DCs that survey the contents at the distal convoluted tubule encounter soon-to-be-excreted low molecular weight antigens at much higher concentrations than systemic circulation. Presentation of these innocuous antigens without inflammatory signals to T cells can potentially root out any autoreactive T cells, meaning that the kidneys assist in sustaining peripheral tolerance to harmless antigens, such as endogenous and food metabolites [2,9]. Besides peripheral tolerance, Mac heterogeneity has large implications for cytokine signaling within kidney health. M1 Mac has proinflammatory signaling and the M2 subtype has anti-inflammatory effects through the production of cytokines IL-10 and TGF-β. M2 Mac has been shown to protect against acute kidney injury (AKI), glomerulosclerosis, tubular atrophy, interstitial expansion and renal fibrosis in mice [10,11,12]. While the M1/M2 Mac ratio is tightly regulated, obesity and chronic inflammation states such as CKD lead to a higher expression of the M1 phenotype and a higher M1:M2 ratio [13,14]. Autophagy is also reno-protective against inflammation, as it prevents the accumulation of mitochondria, lysosomes and Damage-Associated Molecular Patterns (DAMPS) and suppresses inflammasome activation [15].

### 2.2. Why the Kidney Is Vulnerable to Inflammation

While the kidney has some protective effects against systemic inflammation, the kidney, as displayed in CKD and many other renal pathologies, is highly susceptible to damage from proinflammatory cytokines and oxidative stress. The kidney has a unique pairing of traits, making it particularly vulnerable to inflammation. The kidneys receive 25 percent of the entire blood volume without having anti-inflammatory defenses found in other highly vascularized organs, such as antioxidants or detoxifying agents of hepatic tissue. Damage from the physiologic hypoxic environment present within the medulla is prevented by regulatory hormones and vasoactive molecules, which are disrupted during inflammation. Furthermore, intrarenal changes in microvasculature triggered by chronic inflammation result in renal damage [2]. Furthermore, the renal tubules are home to many inflammatory cytokines, chemokines and mediators of fibrosis, which are key in responding to renal insults and injury. These markers are highly regulated; however, dysregulation contributes to maladaptive response and repair and progression to CKD. Dysregulation can occur due to exposure to repeat insults, such as diabetes, incomplete recovery from AKI and uncontrolled inflammatory responses [16]. In addition, the proximal tubules have high energy demands, making them prone to ischemia during inflammation and other times of energy supply-demand mismatch [17]. Oxidative damage is also increased in CKD, secondary to increased reactive oxygen species (ROS) and decreased nitric oxide due to increased homocysteine. Furthermore, antioxidant systems such as the glutathione redox cycle are at capacity, with an increased oxidized to reduced glutathione ratio, resulting in decreased ability to combat oxidation in CKD patients [6]. In patients with CKD, inflammatory markers IL-1—which is elevated in dialysis patients—fibrinogen and TNF-α are independent predictors of CKD progression [16,18]. Inflammatory activation in CKD also appears to be influenced by genetic and epigenetic conditions [3].

### 2.3. Endothelial Injury

The microvasculature of the kidney operates in an extreme environment with vastly changing levels of oxygenation and osmolality. Therefore, it is tightly regulated by vasoactive molecules, such as prostaglandins, endothelins, kinins, medullipin, nitric oxide and others, in order to keep the corticomedullary osmotic gradient intact, and any alteration to this delicate balance renders the kidneys vulnerable to dysfunction and damage [2]. One factor to consider in this microvasculature damage is renal vasculature resistance (RVR) increases. Any inflammatory damage to any portion of the renal microvasculature can increase RVR, causing ischemic kidney damage [19,20]. Any damage to pericytes, such as AKI-induced sepsis or ischemia-reperfusion, causes the pericytes to detach from peritubular capillaries and differentiate into myofibroblasts [21,22]. This has two pathologic effects on the kidney: fibrosis from the newly formed myofibroblasts and the leaky endothelium cascades inflammatory and oxidative damage in the nearby tissue [23]. In CKD patients, elevated levels of inflammatory signals (TNF-α, IL-6, IL-8) and elevated levels of vascular cell adhesion molecule, E-selectin and intracellular adhesion molecule (ICAM) were associated with higher incidences of salt and water retention and disturbances in macro-microcirculation [24].

### 2.4. Role of Different Inflammatory Markers in the Kidney

The renal system has many exposures to inflammatory markers during times of infection. In pyelonephritis, proinflammatory cytokines, such as Tumor Necrosis Factor-α (TNF-α), Monocyte Chemoattractant Protein-1 (MCP-1), Interleukin-6 (IL-6), IL-8 and IL-23, play an important role in mounting the immune response [25,26]. The initial response to injury or infection is similar in the kidneys to the rest of the body. During infection, IL-1 activates adhesion molecule expression in endothelium and induces another chemokine expression to recruit white blood cells (WBCs). TNF-α also activates endothelial inflammatory responses and causes capillary leakage for incoming immune cells. Incoming immune cells (monocytes and macrophages) are attracted by MCP-1. Elevated IL-6 levels confer a fever and acute phase protein response during this time. Neutrophils are chemoattracted by IL-8, and IL-23 upregulates the proliferation of Th17 cells, which incites more proinflammatory responses [27].

While these proinflammatory cytokines have a role in protection, they can also have damaging effects on the kidney. For example, children with acute pyelonephritis, IL-1β and IL-6 were associated with higher incidences of renal scarring [28]. In low-grade chronic inflammatory conditions such as CKD, the persistent release of these cytokines can cause arteriole fibrosis and exacerbate renal injury. Furthermore, TGF-β and IL-1β are secreted by macrophages during low-grade inflammation, resulting in renal fibrosis [28]. T cells have also recently been implicated in inducing renal fibrinogenesis through the IL-23/IL-17 axis [29].

## 3. Inflammation and CKD

### 3.1. Pathophysiology of Inflammasomes

Inflammasomes are protein complexes of the innate immune system responsible for sensing pathogen-associated molecular patterns (PAMPs) and damage-associated molecular patterns (DAMPs) and activating downstream signals via the activation of protease caspase 1. Activation of caspase-1 leads to proteolytic activation of Interleukin-1β and IL-18 and pyroptosis, an inflammatory cell death process. PAMPs constitute bacterial, viral or fungal markers such as flagellin, HepC protein or Beta-glucan. DAMPs are endogenous signals released during tissue injury, including ion efflux, mitochondrial dysfunction and reactive oxygen species [30]. Toll-like receptors (TLRs), C-type lectin receptors (CLRs), intracellular Nod-like receptors (NLRs) and retinoic acid-inducible gene (RIGs) receptors constitute different families of pattern recognition receptors (PRRs), which are responsible for sensing PAMPs and DAMPs [31]. Intracellular molecular patterns are recognized by NLRs and RIGs; extracellular PAMPs and DAMPs are recognized by CLRs and TLRs [32]. PRRs can recruit inflammasome complexes. There are 5 traditional PRRs known to assemble into inflammasomes. Nucleotide-binding oligomerization domain, leucine-rich repeat-containing protein (NLR) receptors such as NLRP1, NLRP4 and NLRC4 and the remaining two pyrin and absent in melanoma 2 (AIM2) are well characterized. However, there are less well-characterized receptors such as NLRP6, NLRP7, NLRP12 and RIG-1. All these inflammasomes activate caspase 1 (canonical pathway) or caspase 11 (non-canonical pathway). The NLRP3 inflammasome has been implicated in the pathophysiology of many renal diseases [33]. Its assembly depends on NEK7, a serine and threonine kinase, for proper NLRP3 oligomerization. The NLRP3 mechanism of activation is still unclear. However, there are several proposed triggers. NLRP3 is triggered by many pathogens, particulate matter, mitochondrial DNA or potassium efflux. As NLRP3 inflammasome lacks CARD (caspase recruiting domain), an adaptor molecule of ASC (apoptosis-associated speck-like protein containing CARD) is needed to activate caspase. Activating caspase results in the proteolytic activation of proinflammatory cytokines such as IL-1β and IL-18 and pyroptosis, an inflammatory cell death process. There are many internal regulators of inflammatory assembly, particularly at the interaction of ASC with caspase through CARD-only proteins (COPs) and PYD-only proteins (POPs), thereby preventing caspase activation. Protein phosphorylation and ubiquitylation also inhibit the activity of ASC and inflammasomes [31].

### 3.2. Inflammasomes in CKD

Some evidence points to the IL-1β/IL-18 axis’ role in the progression of CKD. Specifically, levels of IL-18 might be associated with monocyte chemoattractant protein-1 (MCP-1), which is independently related to the estimated glomerular filtration rate (eGFR). Furthermore, IL-18 is associated with vascular inflammation and calcification in CKD [2]. One study reported a reduction in tubular injury, inflammation and fibrosis after UUO (unilateral urethral obstruction), associated with a reduction in caspase-1 activation and maturation of IL-1β and IL-18 in NLRP3−/− mice [34]. In diabetic nephropathy, NLRP3 inflammasome activation has been reported and NLRP3 or caspase-1 deficiency improved albuminuria and the fractional mesangial area in diabetic mice [35]. Another study used the same UUO model to analyze early renal edema and vascular permeability and found that NLRP3-deficient (−/−) mice led to increased vascular leakage and interstitial edema. They displayed no effect on inflammation and fibrosis [36]. Despite these findings, the relationship between the inflammasome and CKD remains under debate. In addition to inflammasomes, regulating inflammatory markers is a key process mediating the progression of CKD. Nuclear factor kappa beta and nuclear factor erythroid 2 like 2 (Nrf2) are transcription factors that play a role in inflammatory marker regulation. Nrf2, in particular, has an anti-inflammatory role. Numerous antioxidant enzymes are produced, such as glutathione peroxidase, superoxide dismutase, catalase and heme oxygenase −1. In CKD, Nrf2 expression is downregulated.

Furthermore, patients on hemodialysis also have decreased expression of Nrf2. Downstream effects of this decreased expression are increased renal fibrosis, tubular damage, and worsening of CKD. Furthermore, increased mitophagy and mitochondrial biogenesis and regulation of glucose and lipid metabolism are also controlled by Nrf2 in macrophages during infections, helping restore normal cellular metabolism from the glycolysis and lipid synthesis-dominant metabolism during inflammation. Due to decreased Nrf2, defective mitophagy and ROS overproduction, CKD disrupts this process [37].

### 3.3. Role of Leptin in the Development of CKD

Leptin is an anorexigenic molecule in adipose tissue that controls appetite and body mass. However, leptin also has many other effects and regulates functions such as immune and endocrine response, sexual maturation, bone mass, hematopoiesis and blood pressure. It has been involved in the development of CVD and HTN [38]. Leptin levels are often elevated in CKD due to a reduction of normal physiologic clearance performed by megalin-mediated metabolic degradation in the proximal tubules, resulting in a proinflammatory uremic state. Leptin clearance is only effective for hemodialysis patients with high-flux membranes. In addition to poor clearance, hyperinsulinemia and low-grade inflammation have also been implicated in increased leptin levels in CKD [39]. The increased levels of leptin in turn increase the progression of CKD via increased glomerulosclerosis.

Leptin receptors (which stimulate Janus kinase/signal transducers) are mainly present in the inner medulla and vasculature of the cortico-medullary regions [40]. Leptin promotes endothelial cell proliferation by upregulating TGF-B1 levels. TGF-B1 levels induce profibrotic changes and decreased breakdown of the extracellular matrix. More specifically, leptin was not found to increase TGF-1 in the mesangial cells but increased TGF-2 receptor expression in the mesangium [41]. Leptin also increases collagen type 1 generation and glucose uptake [42].

Interestingly, leptin and TGF-1 addition enhance mesangial collagen type 1 generation, suggesting leptin-induced TGF-2 receptor upregulation enhances the interaction with increased TGF-1 levels. This in turn results in glomerulosclerosis and the progression of CKD. Furthermore, increased leptin-mediated sympathetic nervous system activation may promote arteriosclerosis and CKD progression in the kidneys via hypertension [41]. Increased matrix proliferation, neovascularization via vascular endothelial growth factor, promotion of vascular calcifications through vascular smooth muscle cell differentiation to osteoblast cells and prothrombotic effects of leptin all contribute to increased hypertension [40]. Furthermore, leptin was shown to be associated with elevated CRP levels and insulin resistance, which are both linked to CKD. The leptin receptor imitates the gp120 family of cytokine receptors (including the IL-6 receptor), increasing serum CRP levels [43]. Leptin’s proinflammatory effects also increase cytokines such as TNF-alpha, IL-1, IL-2, IL-2 and MCP-1, leading to accelerated atherosclerosis, insulin resistance and endothelial dysfunction [40]. While leptin may promote inflammatory cytokines and CD4+ T cell proliferation, it is associated with interference of innate immunity by inhibiting neutrophil chemotaxis and reducing oxidative burst, resulting in an increased risk of infections in the CKD patient [40].

### 3.4. Metabolic Syndrome and CKD

Metabolic syndrome (MetS) involves insulin resistance, central obesity, hyperglycemia, hyperuricemia, hyperlipidemia and hypertension, and is a risk factor for cardiovascular disease and pathologies such as myocardial infarction, stroke and thrombosis. The estimated worldwide prevalence of MetS is 20–25%, and it is well known that MetS harms the renal system. Several factors influence the pathophysiology of MetS on kidney function, such as obesity, insulin resistance, endothelial dysfunction, obesity, hypertension and inflammation [44].

#### 3.4.1. Insulin Resistance

The cause of insulin resistance is multifactorial. Post-Phosphatidylinositol 2-kinase receptor pathway disruption has been attributed to insulin resistance. Furthermore, poor glucose clearance in CKD, complications of dialysis, obesity, increased leptin: adiponectin ratio, vitamin difference and chronic inflammation all lead to insulin resistance. Insulin resistance mainly progresses to CKD through fibrosis [45]. First, insulin resistance promotes sodium retention, subsequent RAAS activation and lipid accumulation in the tubules. Furthermore, insulin-mediated TGF-1 activation activates mesangial proliferation, fibrosis and progression to CKD. Similarly, insulin resistance causes sterol regulatory element binding protein-1 (SREBP-1) to promote lipid droplet accumulation in renal tubular cells, resulting in further interstitial fibrosis and tubular atrophy. Lastly, the connective tissue growth factor (CTFG) also promotes fibrosis after activation by insulin-like growth factor-1 [46].

#### 3.4.2. Obesity

Obesity has been shown to lead to RAAS activation, elevated aldosterone levels and sodium retention. RAAS activation also results in elevated GFR and renal plasma flow with subsequent glomerular hyperfiltration, glomerulomegaly and segmental sclerosis. Obesity is also associated with elevated leptin levels, contributing to renal damage [46].

#### 3.4.3. Hypertension

Hypertension in MetS is directly related to RAAS activation. Activation of this system is caused by several factors, such as secretion of angiotensinogen by visceral adipocytes, renal parenchymal cell compression by fat buildup around the kidneys with subsequently altered pressure natriuresis and increased sympathetic nerve activity by substances such as leptin. Hypertension directly injures the kidney due to ischemia, which damages the kidney and elevates angiotensin II levels [46].

## 4. CKD-Induced Cardiovascular Disease through Inflammation

CKD plays a key role in the development of cardiovascular disease (CVD) and is a leading cause of death in the CKD population, with mortality risk correlating with decreasing GFR and dialysis [1]. CKD-associated inflammatory markers like IL-1 have been shown to decrease HDL functionality. Interestingly, one recent study has elucidated renal injury-induced lymphangiogenesis in the gastrointestinal tract. More specifically, they found that lymphatic vessel generation was associated with macrophage migration and the protein vascular endothelial factor C (VEGF-C), which renal tubule cells have been documented to activate. Furthermore, CKD-upregulated inflammatory markers such as Il-6, IL-10 and IL-17 have been found in mesenteric lymph, resulting in increased production of IsoLG. Furthermore, CKD induces myeloperoxidase in the intestinal wall, increasing IsoLG generation [47].

CVD has classically been associated with cholesterol as the key agent of its pathogenesis. However, lipid-lowering agents have shown questionable benefits due to CKD-related CVD’s unique complications and pathogenesis [48]. Therefore, some focus has shifted towards inflammation-related lipid peroxidation and resulting prostaglandin H2 production as a cause of CVD. This process leads to the generation of dicarbonyls such as isolevuglandins (IsoLGs) and 4-oxo-nonenal (4-ONE), which are responsible for interfering with cellular and lipoprotein mechanisms through covalent binding. Interestingly, some data support using scavengers of these dicarbonyls to treat atherosclerotic CVD, and even CKD [47].

These dicarbonyls also disrupt normal HDL function, a lipoprotein that has already decreased CKD production [47,49]. The generation of IsoLG also affects the lymphatic system, which affects the cardiovascular system. Inflammation induces the generation of lymphatic tissue and remodeling of the vasculature, which plays a role in the drainage of interstitial fluid and the transportation of cytokines, antigens and other substances. Data have revealed that lymphatic system defects are therefore closely associated with the pathogenesis of atherosclerosis through increased atherogenic lipoprotein levels. Furthermore, lymphatic clearance of dead myocytes and debris is important in tissue repair after cardiac infarction. Data have revealed mice with impaired lymphangiogenesis resulted in having intensified cardiac damage after reperfusion. When examining hypertension and CVD, salt-induced high blood pressure activates lymphangiogenesis as a protective mechanism. Inhibiting this process exacerbates hypertension in the presence of excess salt [47].

## 5. CKD and Inflammatory Biomarkers

There are many standard biomarkers like creatinine and cystatin C for detecting and monitoring glomerular function in CKD. However, there are currently few well-studied markers for inflammatory changes in glomeruli (Table 1). Some studies explore a few markers, such as Kynurenine metabolites, TNFR1 and Kim-1, which are detailed below.

### 5.1. Kynurenine Pathway and CKD

The kynurenine pathway (KP) is the main catabolic pathway for tryptophan (Trp) degradation. It also plays a crucial role in immune regulation by the nicotinamide adenine dinucleotide synthesis pathway [60]. This pathway is also involved in the regulation of homeostasis and pathologic processes [61]. Three large cohort studies have found that CKD onset correlates with increased metabolites of the KP. Kynurenine metabolites are generated from Trp by indoleamine-pyrrole 2, 3 dioxygenase (IDO) activity, which is associated with inflammation in atherosclerosis. One of the metabolites of Trp, 3-hydroxykynurenine, is stimulated by IFN-gamma from macrophages and microglial cells, which causes the upregulation of inflammatory markers like TNF-alpha and IL-6. During persistent inflammation, ROS, uremic toxins and glycation products cause increased IFN-gamma release, resulting in upregulating 3-hydroxykynurenine.

Furthermore, KP metabolites and the Kynurenine: Tryptophan ratio correlate with elevated inflammatory markers in CKD. However, the Kynurenine metabolites have also been shown to induce phenotype switching of T-cells in glomerulonephritis, inducing protection from renal injury. Particularly interesting in CKD is that IDO may confer protection from inflammation-induced renal fibrosis. More research is needed to identify the relationship between kynurenine and immunomodulation in CKD [60].

### 5.2. TNFR1, TNFR2, YKL-40 and KIM-1 in CKD

CKD and advancement to kidney failure with replacement therapy (KFRT) are strongly associated with diabetes, with some individuals experiencing a rapid decrease in eGFR. Disease progression is typically identified by measuring glomerular filtration through serum creatinine and cystatin C-based eGFR. More recently, some focus has shifted to inflammation, fibrosis and tubular health as contributors to CKD progression. One study analyzed these factors in patients with concurrent diabetes and eGFR < 60 mL/min/1.73 m^2^ by measuring levels of circulating tumor necrosis factor receptors 1 and 2 (TNFR1 and TNFR2), chitinase 3-like 1 (YKL-40) and kidney injury marker 1 (KIM1) in comparison with KFRT risk. TNFR1, TNFR2 and YKL-40 are indicators of inflammation and fibrosis, while KIM1 represents tubular injury.

The study found that these biomarkers indicated a higher KFRT risk, regardless of established risk factors such as urinary albumin-creatinine ratio (UACR) and eGFR. The results are partly explained by the activation of the TNF pathway in diabetic nephropathy-induced glomerular injury and endothelial destruction—this activation increased serum levels of TNFR1 and TNFR2. There is less evidence regarding the relationship between serum YKL-40 and renal impairment in CKD and diabetes. However, one study determined higher protein levels were positively correlated with an elevated risk of disease advancement. Notably, these findings were not reproduced by a different study that did not only include patients with an eGFR < 60 mL/min/1.73 m^2^. Thus, this biomarker may better suit individuals with more severe disease manifestations. Lastly, the study above determined serum KIM-1 was correlated with not only KFRT risk, but also the greatest determining biomarker of the group for KFRT. This finding suggests tubular damage may be one of the most influential factors in determining the severity of diabetic nephropathy [62].

### 5.3. Low EGF, High MCP-1 and Alpha-1-Macroglobulin in CKD

It is important to note urinary biomarkers that may point to CKD progression and evaluate the risk of renal function decline in the pediatric population. Although measuring serum biomarkers such as TNFR1, TNFR2, YKL-40 and KIM1 point to tubular injury in CKD, they are influenced by GFR and multi-organ failure. Thus, Greenberg et al. suggest analyzing urinary biomarkers to better analyze tubular injury, remodeling, inflammation and overall health, especially since urinary analysis is non-invasive.

Greenberg et al. investigated the effects of several molecules on CKD progression, tubule health and inflammation, such as epidermal growth factor (EGF), α-1 microglobulin, KIM1 and monocyte chemoattractant protein-1 (MCP-1). The study found that pediatric patients with urinary KIM1, MCP-1 and α-1 microglobulin in the highest quartile and urinary EGF in the lowest quartile were associated with an elevated risk of CKD disease progression [63].

The binding of EGF to the EGF receptor triggers renal tubular cell growth, differentiation and repair, explaining why lowering levels would be associated with the rapid progression of CKD. Furthermore, Azukaitis et al. found that eGFR reduction was associated with lower urinary EGF [64]. KIM1, a protein located in proximal tubular cell apical membranes, is upregulated in the setting of tubule injury and shed into the tubule lumen and subsequent urine. Thus, Greenberg et al. describe KIM1 as being independently positively correlated with CKD disease progression. MCP-1 is involved in the recruitment of monocytes and their conversion to macrophages. This is associated with several renal impairments, such as fibrosis and macrophage recruitment in diabetic nephropathy and eGFR slope in CKD. Moreover, Greenberg et al. revealed a strong relationship between KIM1 and MCP-1 levels (*p* = 0.70) in children with CKD. Lastly, the study found an independent relationship between the progression of CKD in children with glomerular-derived CKD and serum α-1 microglobulin levels. This relationship was only present in patients with eGFR ≥ 60 mL/min per 1.73 m^2^, since α-1 microglobulin is typically filtered by the glomerulus and reabsorbed by PCT cells [63].

## 6. CKD and the Microbiome

### 6.1. CKD and Periodontal Disease

Periodontal disease (PD) encompasses the inflammatory pathologies of gingivitis and periodontitis [65]. Several chronic disorders, including atherosclerotic disease, type 2 diabetes mellitus (TDM), coronary heart disease and stroke, have been linked to PD [66,67]. In PD, there is an imbalanced interaction between periodontal microbiota and the host’s inflammatory response; however, it is unknown how this imbalanced relationship links periodontitis to systemic disease [68]. A growing body of evidence supports the association between CKD and PD. However, the mechanisms by which PD promotes the progression of CKD remain unclear [69,70,71]. It remains to be seen if a dysregulated inflammatory response or the effect of a specific pathogen from the dysbiotic microflora from PD exacerbates CKD [71]. The three microbial species strongly associated with PD are Tannerella forsythia, Treponema denticola and Porphyromonas gingivalis. All correlate with high antibody titers in CKD patients [72,73,74]. *P. gingivalis* causes immune dysregulation by interfering with macrophage signaling and inducing ongoing inflammation. Its fimbriae proteins activate complement receptor 3 on monocytes and cause reduced IL-12 and IFN-gamma production, resulting in poor macrophage function and prolonging inflammation by preventing bacterial clearance [72]. Furthermore, lipopolysaccharides (LPS) of gram-negative bacteria like P. gingivalis induce renal fibrosis by increasing TGF-beta expression in the renal cortex. It is believed that toll-like receptor signaling inflammatory pathways activated by LPS affect the kidneys through M1 and M2 macrophage-mediated production of TNF-α and IL-6 in glomerular endothelial cells, promoting glomerular sclerosis [72,75,76]. An additional important virulence factor in P. gingivalis is a family of cysteine proteases called gingipains. Gingipains cause kidney damage through an inflammatory response involving multiple immune cells, including neutrophils, macrophages, and T cells. Gingipains allow the bacterial immune system to avoid phagocytosis by engaging the toll-like receptor 2-phosphoinositide-3 kinase signaling pathway. Furthermore, gingipains cause renal fibrosis by inducing Il-17 expression [72].

As previously stated, PD causes a cascade of inflammation resulting in the release of cytokines (TNF-α, IL-1β, IL-6, IL-8, IL-12, IL-17). These proinflammatory cytokines are associated with periodontitis and kidney disease [73]. It is known that the IL-1 gene encodes inflammatory mediators involved in the pathogenesis of periodontitis and CKD [77]. A study investigating inflammatory markers in CKD patients found that plasma concentrations of IL-6 and TNF-α appeared to be more sensitive markers of odontogenic inflammation in CKD patients [78]. In a separate study, there were significantly higher TNF-α and IL-8 levels in gingival crevicular fluid (GCF) sampled from hemodialysis patients than in a set of control patients from a dental clinic [79].

In conclusion, a relationship between PD and CKD appears to exist, which may be intertwined through various inflammatory mechanisms. However, despite the growing body of evidence suggesting the dysregulated immune response to pathogenic oral bacteria, more investigation is needed to determine if CKD in patients with PD is due to a systemic dysregulated immune response or a result of specific pathogenic oral bacteria causing the release of cytokines by immune cells in the blood.

### 6.2. CKD and Gut Flora

The human microbiome consists of trillions of microbes, many of which live in the gastrointestinal system. The composition of this microbiota is a key factor in determining an individual’s health. A bidirectional relationship between the microbiota and the body depends upon lifestyle, diet and genome. The microbiota exerts many effects on the body and has complex roles in immunity. For example, the microbiota’s short-chain fatty acids (SCFAs) have downstream anti-inflammatory effects through regulatory T cells. Furthermore, SCFAs have also been shown to control blood pressure via olfactory receptor 78-induced renin release (causing hypertension) and GRP41 and GRP43 (causing decreased blood pressure).

Furthermore, the gut microbiome plays a crucial role in maintaining the intestinal barrier to prevent foreign pathogens, toxins and dangerous metabolites from entering the body [80]. Therefore, dysbiosis can occur when this microbiome is disrupted by external environmental factors, host immune response, diet or psychological factors. For example, augmented intestinal oxygen concentrations result in mucus lining changes. Under normal physiologic conditions, the digestive tract is colonized by many obligate anaerobes and fewer facultative aerobes. With dysbiosis, the normally low oxygen concentration increases, resulting in higher numbers of facultative aerobes than anaerobes and decreased activation of MUC genes via hypoxia-inducible factor-1. This results in decreased mucus production lining the epithelial membrane, resulting in susceptibility to pathogens [81]. Dysbiosis therefore results in the overgrowth of harmful pathogens, which produce virulence factors that prevent their clearance and induce the production of uremic toxins that get absorbed through the intestinal barrier and are eventually filtered by the kidneys, a disrupted process in CKD. The uremic toxins also trigger systemic inflammation [80]. Various uremic toxins have negative downstream effects. For example, CKD patients have been found to have increased concentrations of trimethylamine-N-oxide, which correlates with increased mortality risk, atherosclerosis and heart failure [82]. Another uremic toxin, indoxyl sulfate, is linked to ROS production and increased TGF-beta production, causing renal fibrosis [83]. Chronic inflammation also occurs secondary to macrophage migration triggered by pathogen virulence factors like LPS, bacterial DNA and endotoxins. This results in vascular calcification, muscle wasting and insulin resistance.

CKD also causes dysbiosis, and is affected by dysbiosis in many ways. For example, one of the kidney’s main functions is the clearance of nitrogenous waste via the urea cycle—in fact, it handles 80% of the urea clearance. The remaining 20% is carried out by gut flora. In CKD, due to decreased kidney function, much of the urea clearance is carried out by gut flora, resulting in increased generation of ammonium hydroxide and increased intestinal pH [81]. This causes a change in the composition of gut flora, so more pathogenic bacteria thrive, increasing inflammatory insults.

The gut microbiome is a crucial player in maintaining many body functions and is closely tied to the functions of the immune, renal and cardiac systems. Disruption in the composition of this microbiome results in many downstream negative effects, such as atherosclerosis and renal fibrosis.

## 7. Dialysis and Inflammation

Patients experiencing chronic renal failure go on hemodialysis (HD) or continuous peritoneal dialysis (CAPD) while waiting for renal transplantation. Often, HD and CAPD result in alterations in several inflammatory markers. Fibroblast growth factor 23 (FGF23), indoxyl sulfate, sclerostin, alpha klotho, Procollagen 1 N terminal Propetide and beta-CrossLaps are upregulated during dialysis [84]. Indoxyl sulfate is positively correlated with mortality. Oncel et al. found elevated IL-6, TNF-α, high-sensitivity C-reactive protein (hs-CRP), ghrelin and leptin in patients with chronic renal failure on HD or CAPD [85]. IL-6, IL-1β and TNF-α were more elevated in HD patients than in CAPD. A different study found that patients with HD and CAPD had similar TNF-α, IL-10 and IL-6 levels [86]. Oncel et al. found no significant differences in hsCRP between the HD and CAPD groups. One hypothesis for differences in cytokine levels in HD compared to CAPD may be attributed to synthetic membranes and invasive arteriovenous fistula in HD compared to the natural membrane (peritoneum) used in CAPD [85].

## 8. The Interplay between Inflammation, Malignancy and CKD

Over the past decade, there have been advances in understanding the inflammatory microenvironment of malignant tissues. The advance in understanding is partly due to epidemiological studies identifying chronic infection and inflammation as risk factors for cancer. However, while epidemiological studies provided a working hypothesis for the role of inflammation in tumorigenesis, they do not conclude causal relationships or mechanistic links [87].

Cancer development is accepted as a multi-step process in which cells possess genetic alterations that can promote the transformation of phenotypically normal cells to malignant ones. The six hallmarks of malignant growth are: self-sufficiency of growth signals; insensitivity to anti-growth signals; escaping from apoptosis; unregulated proliferation potential; enhanced angiogenesis; and metastasis. Various signaling processes accomplish these shifts within neoplastic cells, and investigations have established that inflammation may play a role in forming the classic evolutionary advantageous hallmarks of malignancy [88].

In addition to accumulating mutations and genomic instability, inflammation establishes a cytokine and chemokine network that may contribute directly to malignant progression. Investigations into the specific roles of key regulatory molecules involved in this process demonstrate that TNF, IL-1 β, IL-6, IL-10 and chemokines may be crucial regulators linking inflammation to cancer [88].

TNF is a multifunctional cytokine secreted by inflammatory cells. It has diverse roles in cell survival, proliferation, differentiation and death. The biological actions of TNF function through two distinct signaling pathways, nuclear factor-κB (NF-κB) and c-Jun N-terminal kinase (JNK). NF-κB promotes cell survival by promoting antiapoptotic pathways. On the other hand, JNK signaling leads to cell death. The crosstalk between NF-κB and JNK ultimately determines the cellular outcome in response to TNF. This dual function of TNF creates a dynamic cellular tug of war that could be either pro- or anti-tumorigenic [89].

Tumor cells are known to express IL-10, IL-6 and IL-1 β. IL-10 is traditionally considered an anti-inflammatory cytokine due to its inhibitory action on the immune response through its inhibitory action on T helper cells’ cytokine production. Through the inhibitory action of IL-10 on the immune system, tumor growth is facilitated [90]. IL-6 is believed to play essential roles in tumor behavior by influencing cell survival and proliferation, migration and invasion, angiogenesis and metastasis [91]. IL-1β is abundant in the tumor microenvironment, promoting tumor growth by recruiting monocytes. Monocytes infiltrate the tumor and differentiate into tumor-associated macrophages (TAMs), which secrete IL-10 into the tumor microenvironment, creating a suitable environment of immunosuppression for tumor progression. IL-1β may act as the master cytokine in tumor progression [92].

Inflammation and tumorigenesis share the goals of maximizing cell proliferation and angiogenesis. The mechanisms of inflammation ultimately contribute to tumorigenesis by promoting the tumor cells to evade the host immune response, resulting in tumor growth, angiogenesis, metastasis and invasion. Moreover, examining the mechanisms at play, two separate pathways (extrinsic and intrinsic) draw on inflammation to promote cancer development. The extrinsic pathway engages inflammation to alter the tumor microenvironment that fosters the six mentioned hallmarks of cancer. The intrinsic pathway involves inflammation driving genetic modifications such as oncogene activation and tumor suppressor gene inactivation [2].

CKD is characteristic of low-grade chronic inflammation, with upregulated expression of many inflammatory markers explained above. There is evidence that CKD is associated with renal cell carcinoma (RCC). There is a bidirectional relationship between CKD and RCC, as decreasing renal function is linked to a higher risk of developing CKD and RCC affects renal function, accelerating decline [93]. Unfortunately, many medical therapies for RCC, such as immune checkpoint inhibitors and vascular endothelial growth factor inhibitors, can also lead to CKD [94]. To discover novel therapeutic approaches, more investigation is needed to decipher the underlying disease mechanisms influencing the association between CKD and RCC and vice versa.

## 9. CKD Management

### 9.1. Impacts on Prognenses

Ebert et al. explain premature aging as a consequence of inflammation on CKD prognosis and management. One key mediator is the promotion of cellular senescence by NF-kB. NF-kB is upregulated by the transcription factor GATA4, resulting in the senescence-associated secretory phenotype (SASP) involving proinflammatory cytokines and chemokines. Premature aging in CKD is also attributed to increased cellular replication from inflammation with subsequent telomere shortening. Furthermore, lifestyle features such as poor diet, smoking and stress, as well as CKD-induced oxidative stress, may contribute to inflammation-mediated telomere attrition [95,96].

Maraj et al. found that physical activity was positively correlated with inflammatory biomarkers (albumin, albumin and CRP) in hemodialyzed patients with CKD. Notably, this relationship was dependent on the age of the patient. The study explained that more data are required to decipher the mechanism behind this finding. The diet should also be considered in this setting. Krishnamurthy et al. found that dietary fiber positively correlates with decreased inflammation and mortality. In fact, St-Jules et al. described the harmful effects of limiting plant-derived foods from the diet. An increase in fiber consumption benefits the gut microbiota, reduces metabolic acidosis (due to the basic pH of plant foods) and can decrease proinflammatory cytokine levels [97,98].

Inflammation in CKD leads to a reduction in iron availability through elevated hepcidin and ferritin levels. Hepcidin blocks iron absorption and efflux through its binding to the ferroportin channel. More specifically, this results in iron accumulation within macrophages and hepatocytes, and subsequent iron deficiency anemia. Thus, therapeutic intravenous (IV) administration of iron should be increased to achieve appropriate hemoglobin levels. On the other hand, Jairam et al. demonstrated that IV iron administration may exacerbate inflammation in ESRD patients, complicating therapeutic recommendations [99,100,101].

### 9.2. Treatment

There are many treatment options for treating CKD (Table 2). The treatments target the array of factors that lead to inflammation, which were discussed above.

#### 9.2.1. Statins

There are many treatment options for treating CKD (Table 2). Statins are one such treatment. There is a strong relationship between CKD and dyslipidemia, involving increased triglycerides (TGs) and low-density lipoprotein (LDL) cholesterol with decreased high-density lipoprotein (HDL) cholesterol. In fact, Kuznik et al. discovered a positive correlation between the progression of dyslipidemia and CKD, with 45.5% of patients having dyslipidemia with stage 1 CKD and 67.8% with stage 4. CKD impacts lipid handling in several ways, such as defective clearing of triglyceride-rich lipoproteins, impaired activity of hepatic triglyceride lipase and lipoprotein lipase, structural alterations in the LDL receptor and reduced production of apo-AI. HMG-CoA reductase inhibitors, also called statins, are widely accepted as the first-line treatment for hyperlipidemia [112].

Furthermore, Wang et al. revealed a therapeutic effect of statins on inflammation associated with CKD. More specifically, they demonstrated CKD patients who took statins had decreased serum c-reactive protein (CRP) levels, an inflammation biomarker. The study also found decreased CRP levels in both predialysis patients and those concurrently on dialysis [103].

The mechanism of statin-induced inflammation reduction in CKD is thought to involve preventing NF-κB accumulation, a mediator of inflammation in CKD. Normally, NF-κB stimulates downstream signaling, which results in proinflammatory cytokines. Another proposed mechanism of action of statins in CKD involves the inhibition of the NLPR3 inflammasome and subsequent IL-1β maturation in mononuclear cells. Statins can also shift macrophage differentiation from the M1 subset (inflammation associated with inflammation) to the M2 subset (anti-inflammatory). Moreover, the medication has increased catalase and superoxide dismutase activity, decreasing ROS levels. It can also inhibit Rac isoprenylation and subsequent NADPH oxidase activity and upregulate NO production, all contributing to reduced ROS levels. Finally, statins can reduce macrophage and T-cell activation with subsequent reduction in the inflammatory response [103,112].

#### 9.2.2. TNF-Alpha Inhibitors

CKD is associated with an increase in inflammation and cytokines such as TNF-α. This inflammatory increase leads to the progression of CKD and renal injury. Etanercept, a TNF-α inhibitor, is an approved therapy for autoimmune diseases. However, its use in CKD has not been widely investigated. Mendieta-Condado et al. analyzed the effects of Etanercept on rats with adenosine-induced CKD. The study observed a significant decrease in serum TNF-α levels within 2–4 weeks of Etanercept administration.

Furthermore, they found improvements in BUN levels, serum urea and serum creatinine in the mice with progressive renal function deterioration after 4 weeks of etanercept administration. The study also looked at the histological and structural morphology of the kidneys in these mice. It was found that etanercept therapy diminished structural changes, interstitial fibrosis, tubular atrophy and inflammatory infiltrate after two weeks, caused by adenosine administration. Etanercept was unsuccessful in preventing renal fibrosis that occurred 4 weeks into adenosine administration. Thus, more investigations need to be conducted to determine the effectiveness of TNF-α inhibitors on CKD [104].

#### 9.2.3. Omega-3 Polyunsaturated Fatty Acids

Discussing the effects of omega-3 polyunsaturated fatty acid (PUFA) supplementation on inflammation is important. A diet high in omega-3 PUFA includes oily fish, nuts and seeds. There are three primary forms: alpha-linolenic acid (found in seeds and nuts), eicosapentaenoic acid, and docosahexaenoic acid (found in fish, algae and phytoplankton). Baggio et al. describe the benefits of this diet in preventing renal inflammation and fibrosis by decreasing oxidative stress, endothelial adhesion molecules and cytokines involved in inflammatory pathways [113].

Li et al. investigated the effect of the omega-3 PUFA diet on mortality in the CKD population. This diet improves inflammation in CKD and aids in blood lipid regulation, such as with low-density lipoprotein, high-density lipoprotein and triglycerides. The study also determined that omega-3 PUFA was associated with an increase in the STAMP2 protein, which resulted in a downregulation of biomarkers of CKD and inflammation [114]. Lee et al. found that the omega-3 PUFA diet promoted antioxidation by regulating the TGF-β/SMAD and Nrf2 pathway in inflammation. Although more research needs to be conducted, omega-3 PUFA supplementation may be a helpful therapy in relieving the harmful inflammation found in CKD [115].

#### 9.2.4. L-Carnitine Supplementation

Carnitine, composed of lysine and methionine, is involved in the trafficking of long-chain fatty acids into the mitochondria as part of the beta-oxidation pathway. Several factors contribute to a decrease in carnitine during dialysis, such as its small molecular size, changes in diet and defective renal carnitine production. This decrease can lead to erythropoietin-resistant anemia, abnormal lipid metabolism, insulin resistance, cachexia and cardiac diseases such as cardiomyopathy and arrhythmias. Thus, carnitine may play a major role in the overall health of patients with ESRD from CKD. Hamedi-Kalajahi et al. explain that L-carnitine supplementation has been well established as helpful therapy for diminishing inflammatory biomarkers in hemodialysis patients. This study also looked at the effect of the supplementation on the pediatric population undergoing hemodialysis. After an intervention, it found lowered serum IL-6 and fasting blood sugar (FBS) with increased free carnitine levels. Notably, CRP levels were not found to be significantly decreased, despite the lower IL-6 levels. Although FBS levels were lowered in the study, data on the effects of L-carnitine on insulin resistance are still inconclusive [107].

#### 9.2.5. Sodium-Glucose Cotransporter Inhibitors

Sodium-Glucose Cotransporter 2 Inhibitors (SGLT2i) are effective agents in slowing the progression of diabetic kidney disease and heart failure. Recently, studies have attempted to explore their efficacy in reducing CKD progression. The Study to Evaluate the Effect of Dapagliflozin on Renal Outcomes and Cardiovascular Mortality in Patients with Chronic Kidney Disease (DAPA-CKD) showed that CKD patients treated with dapagliflozin, an SGLT2i, had reduced hazard ratios for the time to first occurrence of >50% eGFR decline, death secondary to renal or cardiovascular events and all-cause mortality. Therefore, SGLT2i may play an important role in managing CKD [108].

## 10. Conclusions

Inflammation significantly affects CKD’s development, maintenance and exacerbation. A low-grade systemic inflammation exists in the background of CKD, affected by various factors such as uremia, dyslipidemia, metabolic syndrome, intestinal dysbiosis and malnutrition. As stated above, the body’s innate immune response is persistently activated due to these stimuli, resulting in low-grade inflammation that causes various downstream negative effects such as cardiovascular disease and cancer. As stated above, the current literature cites many mechanisms for low-grade inflammation in CKD. However, more research is needed to develop a comprehensive picture of how inflammation develops in and affects CKD. Furthermore, identifying better treatments and diets is necessary to mitigate the potential harms of inflammation in CKD. Furthermore, research is also needed to combat the unique problems posed by renal cell carcinoma, where disease, underlying inflammation and even treatments all further the progression of CKD.

## Figures and Tables

**Table 1:** List of biomarkers and their significance.

Biomarker	Indicator of	Role in CKD	Publication
**Asymmetric dimethylarginine**	Endothelial Dysfunction	Downregulated in CKD, resulting in reduced inhibition of nitric oxide production and endothelial dysfunction	Ravani et al. [50]
**CRP**	Inflammation	CARE trial showed increased concentration correlated with faster functional decline	Fassett et al. [51]
**TNF-alpha**	Inflammation	Plays a role in the upregulation of other inflammatory markers and in renal fibrosis via decreasing pro-apoptotic signals	Taguchi et al. [52]
**Pentraxin-3**	Inflammation	Increases tissue factor expression, associated with increased fibrinogen and part of the early innate immune response, and may play a role in thrombogenesis and vascular ischemia in the kidneys	Tong et al. [53]
**TGF-Beta 1**	Inflammation	Increases extracellular matrix and results in fibrosis of the kidney	Fassett et al. [51]
**Matrix Metalloproteinase-9**	Inflammation	Remodels extracellular matrix, which is a marker for renal fibrosis, may also play a role in increasing hypertension	Catania et al. [54]
**IL-1Beta**	Inflammation	Plays a role in inflammasome formation and initiating the innate immune response	Anders [55]
**IL-6**	Inflammation	It may be involved in atherosclerosis in CKD	Kamińska et al. [56]
**VEGF**	Inflammation	Involved in macrophage recruitment and impaired angiogenesis	Kang et al. [57]
**IL-18**	Inflammation	Triggers upregulation of other inflammatory cytokines and is involved in inflammasome formation	Anders & Muruve [58]
**CSF-1**	Inflammation	Renal macrophage polarization mediated repair in the kidneys	Huen et al. [59]

**Table 2:** List of CKD treatments and their targets and effects.

Target	Treatment	Effect	Study
Lipid metabolism	Omega-3 fatty acids	Increased adipokine signaling resulting in decreased inflammation	Guebre-Egziabher et al. [102]
CRP	High-fiber diet	Decreased CRP and all-cause mortality	Krishnamurthy et al. [98]
Cholesterol metabolism	Statin	Decreased CRP in dialysis and non-dialysis patients	Wang et al. [103]
TNF-alpha	TNF-alpha inhibitor	Possible reduced interstitial fibrosis more data needed	Mendieta-Condado et al. [104]
Immune modulation	Vitamin D	Reduced expression of Il-6, IFN-gamma, TLR7 and TLR 9	Carvalho et al. [105]
Growth Hormone Receptor	Growth hormone	Reduced CRP and homocysteine and increased HDL	Ikizler et al. [106]
Beta oxidation pathway	L-carnitine	Lower IL-6	Hamedi-Kalajahi et al. [107]
Sodium-Glucose Cotransporter 2	SGLT2i	Reduced all-cause mortality	Fernandez-Fernandez et al. [108]
Oxidative Stress	Vitamin E	Reduced VCAM-1 and malondialdehyde and overall oxidative stress	Ngyuen et al. [109]
Gut-flora	Probiotics	Significantly lower levels of inflammation	Wagner et al. [110]
IL-1 receptor	IL-1 receptor antagonist	Lower levels of CRP and IL-6	Hung et al. [111]

## Data Availability

No new data were created or analyzed in this study. Data sharing is not applicable to this article.
